# Surface displaying of swine IgG1 Fc enhances baculovirus-vectored vaccine efficacy by facilitating viral complement escape and mammalian cell transduction

**DOI:** 10.1186/s13567-017-0434-5

**Published:** 2017-05-12

**Authors:** Zehui Liu, Yangkun Liu, Yuanyuan Zhang, Yajuan Yang, Jingjing Ren, Xiaoying Zhang, Enqi Du

**Affiliations:** College of Veterinary Medicine, Northwest A&F University, Yangling, Shaanxi 712100 People’s Republic of China

## Abstract

Baculovirus-mediated gene transfer has been developed as a vaccine design strategy against a number of diseases without apparent viral replication. However, it has been hampered by complement-dependent inactivation, thus hindering the in vivo application of baculovirus. A variety of approaches have been exploited to bypass the complement system in the serum. In this study, we constructed and screened a series of baculovirus vectors displaying complement interfering factors, of which a baculovirus vector displaying swine IgG1 Fc (pFc) showed the highest complement antagonism (75.6%). Flow cytometry analysis of transduced cells demonstrated that the baculovirus display of pFc had a significant increase in transduction efficiency and transgene expression of reporter genes. On this basis, a VSV-G-pseudotyped with swine IgG1 Fc surface displayed baculovirus vector was developed to express the classical swine fever virus (CSFV) E2 gene. The translational enhancers Syn21 and P10UTR were incorporated to improve the antigen expression. The E2 gene was efficiently expressed in both insect and mammalian cells. Pigs immunized with this recombinant baculovirus developed high levels of E2-specific antibody, CSFV-specific neutralizing antibody and IFN-γ-secreting cellular immune responses. These results demonstrate that the strategy of surface-displaying swine IgG1 Fc has a great potential to improve the efficiency of baculovirus-vectored vaccine for CSFV and other swine pathogens.

**Electronic supplementary material:**

The online version of this article (doi:10.1186/s13567-017-0434-5) contains supplementary material, which is available to authorized users.

## Introduction

The baculovirus-based protein expression system has been used extensively to produce a variety of heterogenous proteins in insect cells. Recombinant baculoviruses carrying mammalian cell active expression cassettes, BacMam viruses, have been developed as a vaccine strategy against diseases in several animal models. Intramuscular, intraperitoneal or intranasal vaccination with these recombinant baculoviruses is shown to elicit humoral and cellular immune responses against various antigens [1, 2]. As a gene delivery vector, baculovirus offers several advantages over other viral vectors, including a strong biosafety profile [3, 4], a high capacity for insertion of heterologous DNA, and ease of production. In addition, baculovirus can be used as an adjuvant to stimulate the innate immune system by regulating toll-like receptor 9 (TLR9) and RIG-I-like receptor (RLR) pathways [5]. Therefore, baculovirus is an effective tool for gene delivery.

Baculovirus infects insect cells in nature. The glycoprotein gp64 mediates virus attachment to the cell surface and internalization [6], which comprises an N-terminal signal peptide (SP) and a mature domain that includes the transmembrane domain (TM) and cytoplasmic domain (CTD). After anchoring in insect cells, the gp64 SP directs its transport to the plasma membrane, where gp64 is exhibited on the surface of infected cells as homotrimers. The gp64 CTD interacts with the budding nucleocapsids and directs the incorporation of gp64 into the virion [7]. Thus, foreign proteins coupled to gp64 will be routed efficiently to the cell membrane to be displayed on the surface of infected cells and on baculovirus particles. Recombinant baculoviruses made with this surface display technique have been used in a variety of applications, including functional studies of glycoproteins, drug screening and development of vaccine candidates [8, 9].

Although baculovirus does not suffer from pre-existing antibodies in vertebrates, baculovirus-based gene delivery faces two bottlenecks: complement-dependent inactivation and low transduction efficacy targeting immune cells, such as dendritic cells, macrophages, B cells, and T cells. This has thus affected, to a great extent, the efficacy of baculovirus-vectored vaccines [10]. A number of approaches have been applied to bypass the complement using chemical compounds or complement receptors, and surface display of complement inactivators, such as soluble complement receptor type 1 (sCR1), decay acceleration factor (DAF), membrane cofactor protein (MCP), Smallpox inhibitor of complement enzymes (SPICE) and ornithodoros moubata complement inhibitor (OmCI) [11]. A pseudotyped baculovirus replacing gp64 with vesicular stomatitis virus G (VSV-G) protein has shown improved efficiency in transducing various mammalian cells and antagonism against complement-mediated inactivation [3].

Classical swine fever virus (CSFV) is a causative agent of CSF, a highly contagious Class A infectious disease being classified by the World Organization for Animal Health (OIE), leading to great economic losses in the pig industry [12]. The viral E2 glycoprotein is responsible for eliciting neutralizing antibodies in immunized animals and protecting them from lethal dose challenge. This protein also plays multiple roles in the viral life cycle, and mediating viral entry into host cells [13].

At present, the most extensively used CSFV vaccine is a lapinized attenuated vaccine based on the C-strain. Although it can confer complete protection against CSF, this vaccine does not allow differentiating infection in vaccinated animals (DIVA). Other vaccination strategies, such as avirulent and low-pathogenic viral vectored vaccines expressing E2 protein [14–16], still remain biosecurity concerns.

In this study, we aimed to further improve baculovirus-vectored vaccine efficacy. To explore the effects of complement interfering factor OmCI, SPICE, DAF and pFc displayed on the baculoviral envelope for baculovirus-mediated gene delivery, we constructed four recombinant baculoviruses displaying OmCI, SPICE, DAF and pFc, respectively. We hypothesized that by displaying these elements, the in vitro complement antagonism and transgenic expression of these four recombinant baculoviruses would promote as vaccine delivery vectors. The modified recombinant BacMam carrying CSFV E2 expression cassette under the CMV promoter was tested as a novel CSFV vaccine in generating effective immunity in pigs.

## Materials and methods

### Virus, plasmids, and cells

The CSFV Shimen and CSFV C strains vaccine was purchased from Jilin Zhengye Biological Products Co., Ltd, Jilin, China. The pFc, SPICE, DAF, OmCI and E2 genes were synthesized by GENEWIZ (Suzhou, China). *E. coli* SW106 (with multigene baculovirus expression vector AmMultiBac) was obtained from Professor Yao Lun-guang of Nanyang Normal University, China. Plasmid pFBDM-VSV-ED-CMV-DsRed (containing the CMV promoter and VSVG-ED), swine intestinal epithelial cells (IEC), porcine kidney cells (PK-15) and Sf-9 insect cells were also maintained in our lab. The IEC and PK-15 cells were cultured in Dulbecco modified Eagle medium (DMEM, HyClone, China) supplemented with 10% heat-inactivated fetal bovine serum (FBS, Gibco, CA, USA), 100 units/mL penicillin and 100 μg/mL streptomycin (Invitrogen), and incubated at 37 °C in a humidified 5% CO_2_ atmosphere.

### Construction of baculovirus vectors

**Figure 1:**
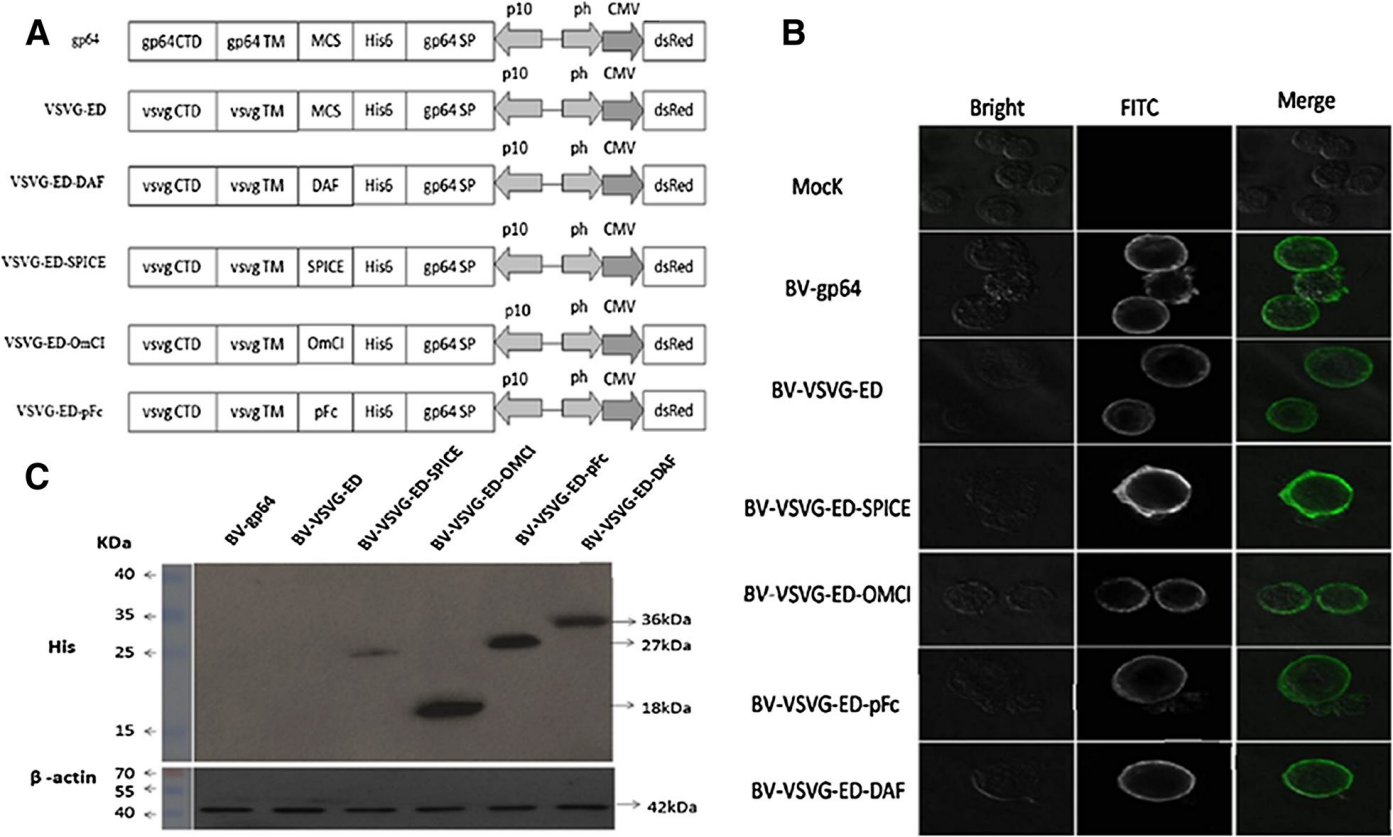
**Identification of recombinant baculoviruses displaying complement interfering factors. A** Schematic diagram of donor vectors of recombinant baculoviruses displaying complement interfering factors on the surface. CMV: human cytomegalovirus (CMV) immediate early enhancer and promoter, ph: AcMNPV polyhedrin promoter, p10: AcMNPV p10 promoter, gp64 SP: Autographa californica nuclear polyhedrosis virus (AcMNPV) gp64 signal sequence, MCS: multiple cloning sites, gp64 TM: AcMNPV gp64 transmembrane domain, gp64 CTD: AcMNPV gp64 cytoplasmic domain, VSVG TM: vesicular stomatitis virus G protein (VSV-G) transmembrane domain, VSVG CTD: VSV-G cytoplasmic domain. **B** Confocal microscopy analysis of His_6_-tagged pFc, SPICE, DAF and OmCI proteins anchoring on the plasma membrane of Sf-9 cells. Protein localization was visualized in cell membrane using a confocal microscope by the primary antibody (anti-His_6_ monoclonal antibodies) and the secondary antibody (FITC-conjugated goat anti-mouse IgG). **C** Western blot analysis of His_6_-tagged pFc (27 kDa), SPICE (26 kDa), DAF (36 kDa) and OmCI (18 kDa) in infected Sf-9 cells.

Six plasmids including pFBDM-VSVG-ED-pFc-CMV-DsRed, pFBDM-VSVG-ED-SPICE-CMV-DsRed, pFBDM-VSVG-ED-DAF-CMV-DsRed, pFBDM-VSVG-ED-OmCI-CMV-DsRed, pFBDM-VSVG-ED-CMV-DsRed and pFBDM-gp64-CMV-DsRed, were constructed by standard methods. A recombinant baculovirus vector vaccine, pFBDM-VSVG-ED-CMV-E2, was constructed to express CSFV E2 gene derived from CSFV strain Shimen (Accession AY775178) under the control of CMV promoter, and pFBDM-VSVG-ED-pFc-CMV-S/P-E2 plasmid was constructed by introducing translational enhancers Syn21 and P10UTR [17], into the above vector (Figure 1). The plasmids were transformed into *E.coli* SW106 competent cells, and recombinant Bacmid (rBac) DNA, including rBac-VSVG-ED-pFc, rBac-VSVG-ED-SPICE, rBac-VSVG-ED-DAF, rBac-VSVG-ED-OmCI, rBac-VSVG-ED, rBac-gp64, rBac-VSVG-ED-CMV-E2, and rBac-VSVG-ED-CMV-S/P-E2, were amplified by PCR amplification using gene-specific and M13R primers.

### Generation and titration of recombinant baculovirus

Sf-9 insect cells were cultured at 27 °C in Sf-900II SFM medium containing 10% FBS (Gibco, CA, USA) and 1% antibiotics (100 U/mL penicillin and 100 μg/mL streptomycin). The cells were transfected with each of the above recombinant Bacmid DNA, and the supernatant were collected at 120 h after cultivation. The viruses were passaged in Sf-9 cells to obtain high titer viral stocks. The culture supernatants from infected cells were collected once 90% of the cells had been infected. The recombinant baculovirus genome was extracted and then checked for the presence of the insert by PCR using gene-specific and baculovirus polyhedron-specific primer pairs. The recombinant baculoviruses were named BV-VSVG-ED-pFc, BV-VSVG-ED-SPICE, BV-VSVG-ED-DAF, BV-VSVG-ED-OmCI, BV-VSVG-ED, BV-gp64, BV-VSVG-ED-CMV-E2 and BV-VSVG-ED-pFc-CMV-S/P-E2, respectively. The titer of the baculovirus stocks was measured by end-point dilution assay. Briefly, Sf-9 cells were plated in 96-well plates, and tenfold serial dilutions of the virus stocks were added to the cells. Viruses and cells were allowed to interact for 1 h before the viruses were removed, and the cells were incubated for 8 days and examined for GFP expression. Wells that appeared green fluorescence were deemed to be baculovirus-positive, otherwise deemed to be baculovirus-negative. Subsequently, the titer for every virus stock was calculated according to the Reed-Muench method.

### Western blot analysis of purified recombinant baculoviruses

The virus supernatant was purified by ultracentrifugation as described before [1]. Briefly, cell debris was first removed by centrifugation for 10 min at 2000 rpm. Infected cell supernatant was then layered over 27% sucrose and centrifuged at 24 000 rpm for 75 min in SW28 tubes. The virus pellet was resuspended in phosphate-buffered saline (PBS, pH 7.5) and centrifuged in SW28 tubes at 27 000 rpm for 150 min. The final pellet was resuspended in PBS. The incorporation of His_6_-tagged pFc, SPICE, DAF, and OmCI into baculoviruses was probed using mouse anti-His_6_ monoclonal antibody (Boster, Wuhan, China). The Sf-9 cells infected with recombinant BV-VSVG-ED-CMV-E2 or BV-VSVG-ED-pFc-CMV-S/P-E2 baculovirus vaccine were analyzed by mouse anti-His_6_ monoclonal antibody or rabbit anti-CSFV polyclonal antibody, followed by confirmation of the expression of pFc and E2.

### Indirect fluorescent assay (IFA)

The IEC cells were collected at 48 h post-transduction, fixed with 4% paraformaldehyde for 20 min at room temperature, and then exposed to rabbit anti-CSFV polyclonal antibody for 1 h at 37 °C. After three washes with PBS, the cells were incubated with Alexa 594-conjugated goat anti-rabbit IgG (Invitrogen, Carlsbad, CA, USA) for 1 h at 37 °C. Fluorescence images were collected using an inverted fluorescence microscope.

### Confocal microscopy

The Sf-9 cells were cultured on sterile cover slips and infected at a multiplicity of infection (MOI) of 10. Two days after infection, the cells were fixed by methanol/acetone (1:1) for 5 min at **−**20 °C, rinsed with PBS, and blocked with 5% skimmed milk for 30 min at 37 °C. The cells were then sequentially incubated with anti-His_6_ mouse monoclonal antibody and FITC-conjugated goat anti-mouse IgG antibody (Boster, Wuhan, China) for 1 h at 37 °C, and washed for three times with PBS. Protein localization was visualized using a confocal microscope (LSM 510, Zeiss, Thornwood, NY, USA).

### Complement antagonism of recombinant baculoviruses

One of the major challenges of baculoviruses as gene transfer vectors is inactivation by serum complement. We therefore tested the survival rate of recombinant baculoviruses in the presence of serum. Briefly, healthy pig serum and mice serum were divided into two parts: one part was heat-inactivated at 56 °C for 30 min, another was left untreated. Recombinant baculoviruses were incubated with inactivated serum and untreated serum, respectively. After 60 min incubation at 37 °C, these recombinant baculoviruses were subjected to titration by end-point dilution assay. The survival rates of baculoviruses were denoted as the percentage of vector survival in the indicated sera compared to the corresponding heat-treated sera [18].

### Flow cytometry analysis

The IEC cells were transduced with BV-VSVG-ED-pFc or BV-VSVG-ED at a MOI of 100, and subjected to flow cytometry (FACSCalibur, BD Biosciences, Franklin Lakes, New Jersey) analysis 1 day after transduction. The percentage of cells emitting fluorescence (% dsRed + cells) and mean FI of each sample were measured three times by counting 10 000 cells in each measurement.

### Evaluation of recombinant baculovirus vaccines

Complement antagonism of BV-VSVG-ED-CMV-E2 and BV-VSVG-ED-pFc-CMV-E2 was measured as mentioned above. Transduction efficiency of swine cells with recombinant baculovirus vaccines was also a critical checkpoint. A third passage (P3) of baculoviruses (BV-VSVG-ED-CMV-E2 and BV-VSVG-ED-pFc-CMV-S/P-E2) was transduced with swine IEC using an MOI of 100 for 12 h. The media containing the virus was replaced by fresh DMEM, and the cells were incubated for an additional 48 h before being fixed for IFA analysis as described above.

### Pig immunization and immune response detection

Four week old pigs (CSFV antigen and antibody negative, provided by China Institute of Veterinary Drug Control) were randomly divided into 4 groups (5 pigs per group). The pigs were intramuscularly immunized with BV-VSVG-ED-CMV-E2 and BV-VSVG-ED-pFc-CMV-S/P-E2 baculoviruses (10^9^ PFU), CSFV commercial vaccine C-strain (vaccinated control, 2 mL), and PBS (unvaccinated control), respectively, boosted once at 14 days. The CSFV-specific antibodies were tested in sera collected at 0, 7, 14, 21 and 28 days post-infection (dpi) using commercial ELISA kit (Keqian, Wuhan, China), and IFN-γ was assayed in sera collected at 0, 14 and 28 dpi using a commercial ELISA kit (4A Biotech, Beijing, China). All animal procedures were approved and supervised by the Animal Care Commission of the College of Veterinary Medicine, Northwest Agriculture and Forestry University. Every effort was made to minimize animal pain, suffering and distress and to reduce the number of animals used.

### Serum-virus neutralization test (SNT)

Sera collected at 0, 7, 14, 21 and 28 dpi were tested for neutralizing antibodies as previously described [19]. Briefly, serially diluted sera were mixed with an equal volume of 200 TCID_50_ of CSFV Shimen strain, incubated at 37 °C for 1 h, added to PK-15 cells preplated in 96-well culture plates, and cultured for 3 days. The CSFV neutralizing antibody (NAb) titers were determined and expressed as the reciprocal of the highest dilution at which infection of PK-15 cells was inhibited in 50% of the culture wells.

### Statistical analysis

All experiments were performed with at least three independent experiments. Statistical significance was determined by the Student *t* test when two groups were compared, or by one-way analysis of variance (ANOVA), when more than two groups were compared. *P* value < 0.05 was considered to be statistically significant.

## Results

### Identification of recombinant baculoviruses displaying complement interfering factors

Recombinant BV-VSVG-ED-pFc, BV-VSVG-ED-SPICE, BV-VSVG-ED-DAF and BV-VSVG-ED-OmCI baculoviruses were constructed by cloning pFc, SPICE, DAF or OmCI containing a N-terminal His_6_ and C-terminal VSV-G (Figure 1A). The gp64 signal peptide was aimed at translocating the protein to the insect cell plasma membrane and was cleaved, thus exposing the His_6_ tag to the outer surface. To determine whether the complement interfering factors were properly translocated to the cell surface, the baculovirus-infected cells were collected at 2 days after infection, and subjected to IFA. As shown in Figure 1B, the test proteins localized within the plasma membrane, demonstrating that the complement interfering factors were located on the surface of Sf-9 cells. To confirm the expression of His_6_-tagged pFc, SPICE, DAF, and OmCI, Sf-9 cells were infected with recombinant BV-VSVG-ED-pFc, BV-VSVG-ED-SPICE, BV-VSVG-ED-DAF and BV-VSVG-ED-OmCI baculoviruses, respectively, harvested at 3 days after infection, and used for detection. Western blot analyses showed that the complement interfering factors were correctly expressed (Figure 1C).

### Enhanced complement antagonism and transformation efficiency of recombinant baculoviruses

**Figure 2:**
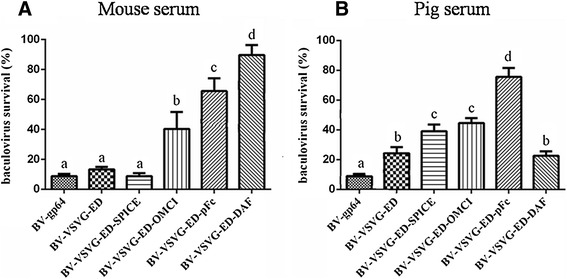
**Evaluation of complement antagonism of recombinant baculoviruses displaying complement interfering factors in serum. A** Baculovirus was incubated with 40% mice serum, and then subjected to titration by end-point dilution assays. Bars denote the percentage of vector survival, which was determined as the percentage of virus titer resulting from pre-incubation with untreated compared to heat-treated serum. Bars with different letters were significantly different (*P* < 0.05). **B** Baculovirus vector survival in pig serum tested as above. Bars with different letters were significantly different (*P* < 0.05).

**Figure 3:**
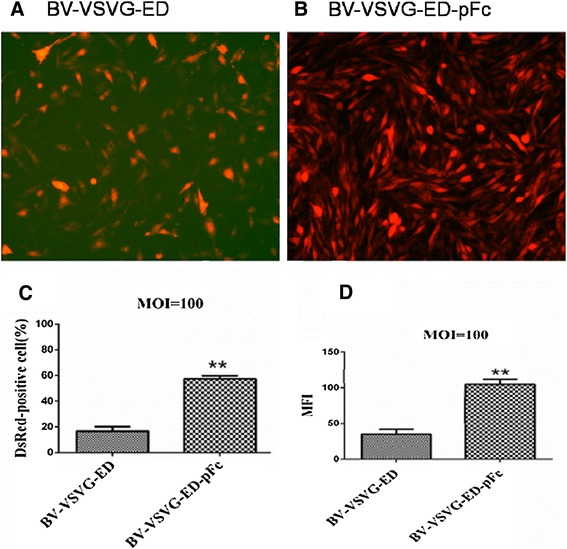
**Surface displayed swine IgG1 Fc enhanced baculovirus-mediated DsRed expression. A**, **B** Fluorescent microscopic analysis of transduced IEC cells 36 h after transduction (×100). **C**, **D** Flow cytometry analysis of transduced cells. **C** Percentage of DsRed positive cells in IEC. **D** Mean fluorescence intensity (MFI) of DsRed positive IEC cells (**P < 0.05).

For application in vivo, recombinant baculoviruses should remain active in the presence of mammal sera. The survival rates of recombinant baculovirus vector were evaluated for complement antagonism. Recombinant BV-VSVG-ED-pFc, BV-VSVG-ED-SPICE, BV-VSVG-ED-DAF, and BV-VSVG-ED-OmCI baculoviruses were treated and titrated as described above. In mouse serum, recombinant baculovirus display of DAF showed a higher complement antagonism activity (90.2%) than recombinant baculovirus display of swine IgG1 Fc (pFc) (65.6%, *P* < 0.05) (Figure 2A). As shown in Figure 2B, in pig serum, recombinant baculovirus display of pFc showed a significantly higher complement antagonism activity (75.6%, *P* < 0.05) than other recombinant baculoviruses. Furthermore, BV-VSVG-ED demonstrated a higher complement antagonism activity (*P* < 0.05) compared with wild type BV-gp64. DsRed expression was analyzed by fluorescence microscopy following transduction of IEC cells with BV-VSVG-ED-pFc or BV-VSVG-ED at an MOI of 100 (Figures 3A and 3B). Transduction efficiency was further quantified by flow cytometry analysis, in which BV-VSVG-ED-pFc demonstrated significantly increased numbers of DsRed positive cells (Figure 3C), and the mean fluorescence intensity (MFI) of DsRed fluorescence was significantly higher with BV-VSVG-ED-pFc than with BV-VSVG-ED (Figure 3D).

### In vitro evaluation of BacMam expressing E2 based on swine IgG1 Fc surface display

**Figure 4:**
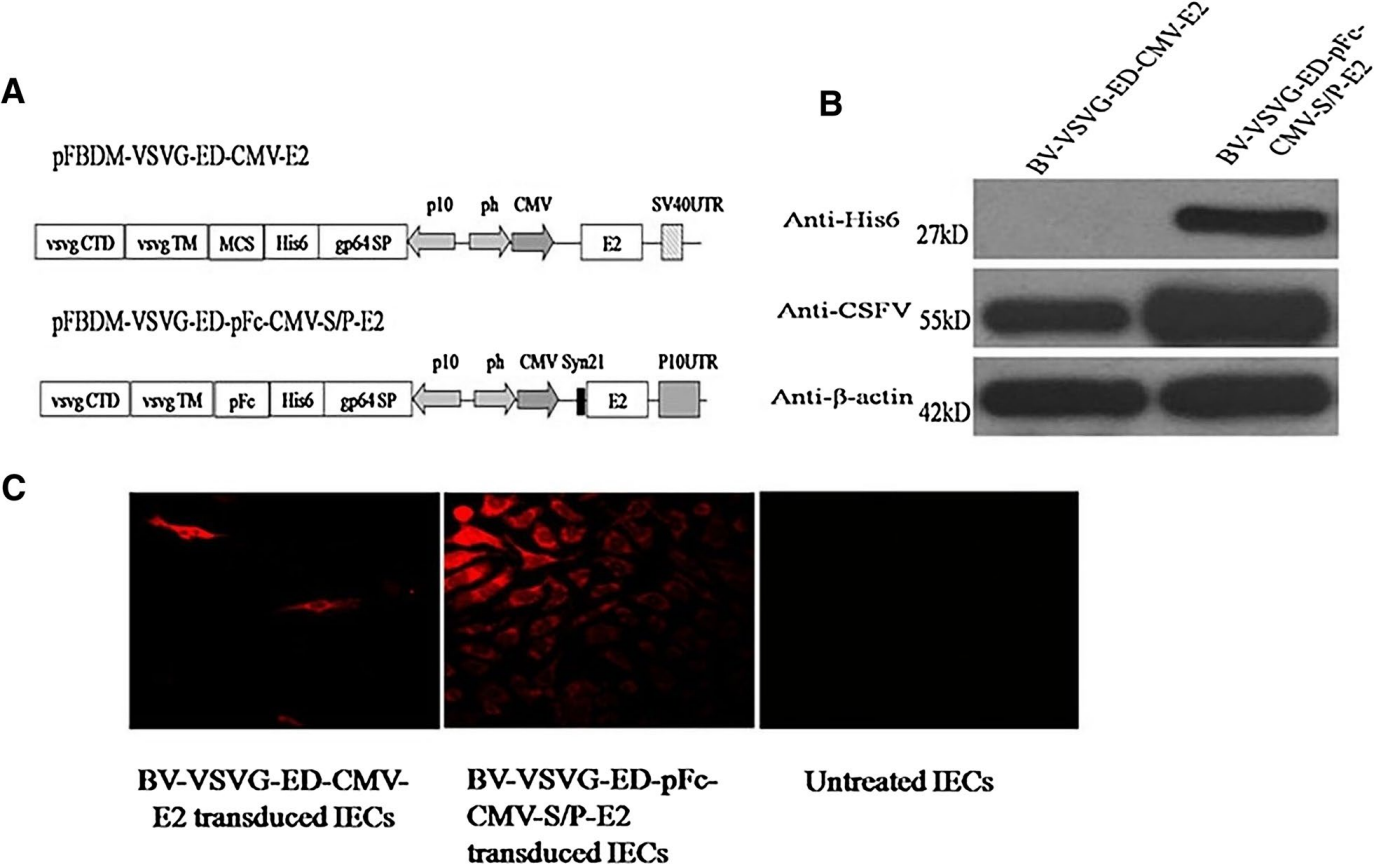
**Characterization and evaluation of BacMam expressing E2 based on swine IgG1 Fc surface display. A** Schematic diagram of donor vectors of BacMam virus vaccines. **B** Western blot detection of pFc and E2 protein expression in insect cells infected with recombinant baculoviruses. BV-VSVG-ED-CMV-E2 or BV-VSVG-ED-pFc-CMV-S/P-E2-infected Sf-9 cells were harvested 2 days after infection and used for analysis. The E2 protein (55 kDa) was detected by rabbit anti-CSFV polyclonal antibody, and His_6_-tagged pFc (27 kDa) was analyzed by anti-His_6_ monoclonal antibody. Anti-β-actin was included as an internal control. **C** IFA detection of expression of E2 protein in BV-VSV-ED-CMV-E2 or BV-VSVG-ED-pFc-CMV-S/P-E2-transduced IEC cells. The cells were fixed at 48 h after transduction, and analyzed by rabbit anti-CSFV polyclonal antibody and Alexa 594-conjugated goat anti-rabbit IgG. Uninfected IEC cells were served as a negative control.

**Figure 5:**
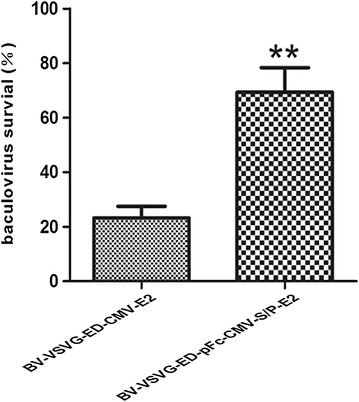
**Complement antagonism of BacMam vaccine vectors in pig serum.** Baculovirus was incubated with 40% pig serum, and then subjected to titration by end-point dilution assay. Bars denote the percentage of vector survival, which was determined as the percentage of virus titer resulting from pre-incubation with untreated compared to heat-treated serum. There was significant difference between BV-VSVG-ED-pFc-CMV-S/P-E2 and BV-VSVG-ED-CMV-E2 (**P < 0.05).

Recombinant BV-VSVG-ED-CMV-E2 and BV-VSVG-ED-pFc-CMV-S/P-E2 baculoviruses were constructed by replacing dsRed with CSFV E2 gene, and incorporating translational enhancers Syn21 and P10UTR to improve E2 expression (Figure 4A). Sf-9 cells infected with recombinant BV-VSVG-ED-CMV-E2 and BV-VSVG-ED-pFc-CMV-S/P-E2 baculoviruses were subject to Western blot analyses (Figure 4B), and it revealed that the E2 and His_6_-tagged pFc protein were correctly expressed in Sf-9 cells. The results from IFA indicated that IEC cells transduced with BV-VSVG-ED-CMV-E2 and BV-VSVG-ED-pFc-CMV-S/P-E2 developed immunofluorescence signals, confirming the expression of E2 protein in IEC cells. Particularly, BV-VSVG-ED-pFc-CMV-S/P-E2 had higher efficiency of gene transduction compared with BV-VSVG-ED-CMV-E2 (Figure 4C). These data were in agreement with the complement antagonism result of BV-VSVG-ED-CMV-E2 and BV-VSVG-ED-pFc-CMV-S/P-E2, which showed that BV-VSVG-ED-pFc-CMV-S/P-E2 resulted in a significantly higher survival rate than that of BV-VSVG-ED-CMV-E2 (Figure 5) (*P* < 0.05).

### Immune responses elicited by BV-VSVG-ED-pFc-CMV-S/P-E2 in pigs

**Figure 6:**
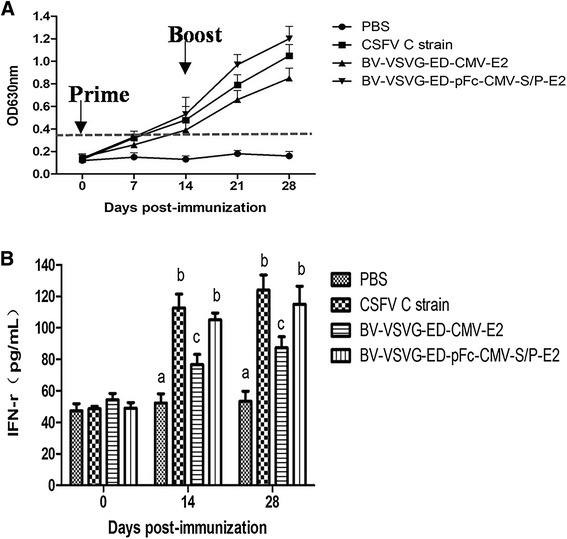
**Detection of antibody and cellular immune responses induced by recombinant baculoviruses. A** Antibody levels in sera of immunized pigs by indirect ELISA. Four groups of pigs were intramuscularly injected twice with commercial vaccine C-strain, BV-VSVG-ED-pFc-CMV-S/P-E2 or BV-VSVG-ED-CMV-E2 at a 1-week interval. Serum samples from each group were collected weekly and tested. A dashed line indicates the positive cutoff value of ELISA (OD630 nm = 0.35). **B** IFN-γ levels in sera of immunized pigs by ELISA. Serum samples from each group were collected and tested using a commercial ELISA kit (4A Biotech, Beijing, China) for quantitative detection of pig IFN-γ. Bars with different letters were significantly different (*P* < 0.05).

To explore the ability of BV-VSVG-ED-pFc-CMV-S/P-E2 to induce CSFV-specific humoral immune responses, pigs were immunized with this baculovirus, as well as BV-VSVG-ED-CMV-E2, commercial vaccine C-strain, and PBS controls, and detected for E2-specific antibodies by ELISA. All the pigs immunized with BV-VSVG-ED-pFc-CMV-S/P-E2, C-strain vaccine, and BV-VSVG-ED-CMV-E2 developed high levels of antibody, without significant difference. The pigs immunized with PBS only demonstrated a non-specific antibody response below the threshold (Figure 6A).

To detect CSFV-specific NAb titers, SNT was performed at 0, 7, 14, 21 and 28 dpi. The results showed that pigs immunized with the commercial vaccine C-strain developed the highest NAb titers, and those immunized with BV-VSVG-ED-pFc-CMV-S/P-E2 also induced considerable level of Nabs, followed by those immunized with BV-VSVG-ED-CMV-E2. Nevertheless, the pigs immunized with PBS did not develop a detectable NAb titer against CSFV even at 4 weeks following prime immunization (Table 1).

**Table 1:** **CSFV-specific neutralizing antibody titers in pigs immunized with BV-VSVG-ED-pFc-CMV-S/P-E2 at different days post-immunization**

Groups	Pig No.	Days post-immunization				
		0	7	14	21	28
		NAbs				
A	039	<1	<1	<1	<1	<1
	075	<1	<1	<1	<1	<1
	086	<1	<1	<1	<1	<1
	116	<1	<1	<1	<1	<1
	200	<1	<1	<1	<1	<1
	$\overline{X}$ ± S	<1	<1	<1	<1	<1
B	022	<1	6	16	64	128
	046	<1	8	16	64	128
	105	<1	6	16	64	128
	142	<1	8	24	80	144
	183	<1	8	16	64	108
	$\overline{X}$ ± S	<1	7.2 ± 1.1	17.6 ± 3.6	67.2 ± 7.2	131.5 ± 12.8
C	035	<1	6	8	16	44
	056	<1	8	8	16	64
	073	<1	6	8	32	64
	117	<1	8	12	16	64
	152	<1	4	8	16	64
	$\overline{X}$ ± S	<1	6.4 ± 1.7	8.8 ± 1.8	19.2 ± 7.2	60 ± 8.9
D	026	<1	6	12	48	84
	084	<1	8	16	48	96
	093	<1	6	12	64	96
	128	<1	8	16	48	100
	192	<1	4	16	64	88
	$\overline{X}$ ± S	<1	6.4 ± 1.7	14.4 ± 2.2	54.4 ± 8.8	95.2 ± 6.6

Four groups (*n* = 5) of 4 week old pigs were immunized with BV-VSVG-ED-pFc-CMV-S/P-E2. Serum were collected at different time after the immunization, and subjected to detection of CSFV-specific neutralizing antibodies (NAbs) by serum-virus neutralization test (SNT). $\overline{X}$ ± S: mean ± standard deviation. Group A-D indicate pigs immunized with PBS, CSFV commercial vaccine C-strain, BV-VSVG-ED-CMV-E2 and BV-VSVG-ED-pFc-CMV-S/P-E2, respectively.

The IFN-γ levels in immunized sera can reflect the immune state of the host. IFN-γ secreted by T-helper type 1 (Th1) cells plays important roles in regulating the cellular immune response. The serum IFN-γ concentrations (14 and 28 dpi) were a little higher in the group immunized with commercial vaccine C strain than the group immunized with BV-VSVG-ED-pFc-CMV-S/P-E2, but not significantly different. Most notably, the IFN-γ level in the group immunized with BV-VSVG-ED-pFc-CMV-S/P-E2 was significantly higher than that of the group immunized with BV-VSVG-ED-CMV-E2 (*P* < 0.05) (Figure 6B) (*P* < 0.05).

## Discussion

Baculoviruses are insect-specific viruses in nature, thus being non-pathogenic for mammals. Baculovirus has been proved to be able to transduce various mammalian cells of human, rodent, porcine, canine, avian, feline and rabbit origins. BacMam viruses are emerging as a gene delivery vector with additional advantages over conventional vectors.

In this study, considering the complement-mediated inactivation of baculovirus, we developed four baculovirus constructs displaying complement interfering factors, and by in vitro evaluation of complement antagonism, the baculovirus displaying pFc achieved a survival rate up to 75.6% in pig serum and 65.6% in mouse serum. Flow cytometry analysis of transduced IEC cells, a cell line with high level expression of Fc receptors, demonstrated that the baculovirus display of pFc showed a significant increase in transduction efficiency (57.5 vs 16.7%) and transgene expression of reporter genes. These results revealed that baculovirus displaying pFc can be used as a promising vaccine delivery vector.

To test the potential of baculovirus displaying pFc as a vaccine delivery vector in vivo, a novel baculovirus vaccine for CSFV was constructed. In terms of optimizing the vaccine constructs, the regulatory elements VSV-G increased antigen presentation, and Syn21 and P10UTR improved antigen expression. Although it did not develop comparative immune response as the commercial vaccine C-strain, this baculovirus vaccine demonstrated a potential for further usage. The high titers of CSFV-specific antibody and neutralizing antibody, and increased levels of IFN-γ secretion indicated that the baculovirus effectively delivered exogenous antigen to the swine cells and stimulated the production of humoral and cellular immune responses. These results suggest a promising vaccine strategy based on the BacMam virus and baculovirus surface display system. It is notable that BV-VSVG-ED-pFc-CMV-S/P-E2 induced stronger CSFV-specific immune response than BV-VSVG-ED-CMV-E2, potentially because it has the translational enhancers Syn21 and P10UTR.

Another important question needing to be addressed is that surface display system-based IgG1 Fc makes a great difference in the efficacy of the BV-VSVG-ED-pFc-CMV-S/P-E2 vaccine. IgG Fc has been intensely investigated as a scaffold for the design of novel therapeutics due to its beneficial biological and pharmacological properties. One of the benefits of Fc-fusion proteins is their interaction with the neonatal Fc receptor (FcRn) on immune cells, thus to increase plasma half-life, and prolong therapeutic activity, providing a new perspective for vaccine development [20, 21]. It has been demonstrated that the Fc part of IgG or IgY (egg yolk antibody) can function as an immunoenhancer [22, 23]. The Fc can also fold in its independent manner and facilitate the stability of the fused proteins. In most cases, the Fc domain forms homodimers with higher affinity for the FcγRs, which enhances binding and the signaling capacity. Takashima et al. demonstrated that pseudorabies virus surface displaying IgG1 Fc gives rise to a better immune efficacy [24]. They also found that vaccination with recombinant cell surface expressing mouse IgG Fc achieves an increase in interleukin, but does not activate the complement classical pathway [25]. Another group has proved that surface display of IgG Fc on baculovirus vectors could enhance the binding to antigen-presenting cells and cell lines expressing Fc receptors [26]. These findings indicate that baculovirus based on IgG Fc surface display can be further explored as a vaccine strategy.

Our study demonstrates that the surface displayed Fc markedly improved the potency of BacMam virus. The molecular mechanism underlying this enhanced efficacy may include the following: (1) prolonging baculovirus-vectored vaccine half-life and promoting effective antigen presentation by interaction with FcRn, (2) using adjuvant function of IgG antibody Fc to activate stronger innate and adaptive immunity by interaction with FcγRs, usually with high level expression in immune cells (dendritic cells, macrophage, and B cells), thus enhancing the signaling capacity for the immune system, and (3) escaping from complement-mediated inactivation in the similar way of Fc-fusion drugs and recombinant cells displaying IgG1 Fc [25, 27–29].

The in vitro studies have proved that baculovirus can bind directly to IgM and C3b in serum, thus activating the classical and alternative pathways [30]. Our previous study has also demonstrated that the complement derived from pig serum inactivated baculovirus by activating the classical and alternative pathways (Additional file 1). Interestingly, we found that BV-VSVG-ED-pFc bound more poorly to IgM in pig serum compared to BV-VSVG-ED, indicating that this surface-displaying swine IgG1 Fc resulted in an increase in complement escape by the classical pathway (Additional file 2). To some extent, this phenomenon was in agreement with previous observations that most Fc-fusion drugs and recombinant cells displaying IgG1 Fc demonstrated remarkable complement antagonism, partially because complement activation depends on the Fab (especially the CH1 domain) and hinge area [31].

In addition to serum complement, baculovirus-specific adaptive immunity also neutralizes the particles and impedes subsequent baculovirus injection in vivo [32]. Given that the BV-VSVG-ED-pFc-CMV-S/P-E2 vaccine was initially tested to evaluate immune responses in pigs with an immune procedure without optimization, though the baculovirus vector surface-displaying pFc potentiated higher level of E2-specific antibody, CSFV neutralizing antibody titer, and IFN-γ than that without pFc surface display, the efficiency of adaptive immune responses was a little lower than the commercial vaccine C-strain. Therefore, it can be improved for better efficacy by using a 3-week immunization interval or adoption of heterologous prime-boost vaccine strategy. Heterologous prime-boost regimens mostly use a viral or DNA vector for priming, followed by a boost with a subunit vaccine. This immunization strategy combines a stronger cellular immune response with a higher antibody response against the vaccine target compared to homologous immunization and can overcome the issue of anti-vector immunity [33].

We aimed to break the bottlenecks limiting baculovirus-based gene delivery for mammals. The swine IgG1 Fc displayed on the BV-VSVG-ED-pFc-CMV-S/P-E2 surface was closely related to the transduction efficiency targeting cells harboring Fc receptors, IEC cells in this study, and it also facilitated baculovirus escape from the complement in pig serum. A more comprehensive understanding of this BacMam based vaccine is essential to improve the immunogenicity of the baculovirus vector. To this end, we are currently focusing on two issues: (1) exploring in more detail the interaction between the baculovirus vector surface-displaying pFc and Fc receptors or elements in the pig serum complement system and (2) testing BV-VSVG-ED-pFc-CMV-S/P-E2 in different immunization schedules to induce stronger immune responses.

In summary, this study provides supporting evidence for the use of the BacMam virus as a vaccine delivery vector. This novel VSV-G-pseudotyped baculovirus based on IgG1 Fc surface display offers an alternative strategy for vaccine development for CSFV and other pathogens.

## Additional files

**Additional file 1.** **Effect of chelating agents on the survival of baculovirus in pig serum.** Baculovirus vectors were pre-incubated with 90% serum for 60 min at 37 °C with or without the addition of a chelating agent, 20 mM EDTA, to chelate Ca^2+^and Mg^2+^, thereby inhibiting all three complement pathways, or 20 mM EGTA/14 mM MgCl_2_ to chelate Ca^2+^, thereby isolating the alternative pathway. The survival of virus was determined by point-end dilution assay on Sf-9 insect cells. Bars denote the percentage of vector survival in the indicated sera referred to the corresponding heat-treated sera. Data represent mean ± standard deviation (SD) of three experiments each of two repeats. Survival in EDTA serum was significantly improved compared with normal serum (*P* < 0.05).

**Additional file 2.** **Detection of interaction between IgM in pig serum and recombinant baculoviruses by ELISA.** 7 × 10^6^ pfu of baculovirus was coated, and then exposed to pig serum, followed by addition of HRP-conjugated goat-anti-swine IgM. Bars denote the OD_450_. The assay was performed in triplicate and the data are presented as mean ± SD (**P < 0.05).
