# Supplementary material for: Surface displaying of swine IgG1 Fc enhances baculovirus-vectored vaccine efficacy by facilitating viral complement escape and mammalian cell transduction
Source: Vet Res. 2017 May 12;48:29. doi: 10.1186/s13567-017-0434-5 (PMC5429525; doi:10.1186/s13567-017-0434-5)
Supplement: Supplementary file 1 — Additional file 1. Effect of chelating agents on the survival of baculovirus in pig serum. Baculovirus vectors were pre-incubated with 90% serum for 60 min at 37 °C with or without the addition of a chelating agent, 20 mM EDTA, to chelate Ca2+and Mg2+, thereby inhibiting all three complement pathways, or 20 mM EGTA/14 mM MgCl2 to chelate Ca2+, thereby isolating the alternative pathway. The survival of virus was determined by point-end dilution assay on Sf-9 insect cells. Bars denote the percentage of vector survival in the indicated sera referred to the corresponding heat-treated sera. Data represent mean ± standard deviation (SD) of three experiments each of two repeats. Survival in EDTA serum was significantly improved compared with normal serum (P < 0.05). [file 13567_2017_434_MOESM1_ESM.doc]

**Fig.S1. Effect of chelating agents on the survival of baculovirus in pig serum.** Baculovirus vectors were pre-incubated with 90% serum for 60 min at 37^◦^C with or without the addition of a chelating agent, 20 mM EDTA, to chelate Ca^2+^and Mg^2+^, thereby inhibiting all three complement pathways, or 20 mM EGTA/14 mM MgCl_2_ to chelate Ca^2+^, thereby isolating the alternative pathway. The survival of virus was determined by point-end dilution assay on Sf-9 insect cells. Bars denote the percentage of vector survival in the indicated sera referred to the corresponding heat-treated sera. Data represent mean ±standard deviation (SD) of three experiments each of two repeats. Survival in EDTA serum was significantly improved compared with normal serum (*P*< 0.05).

**
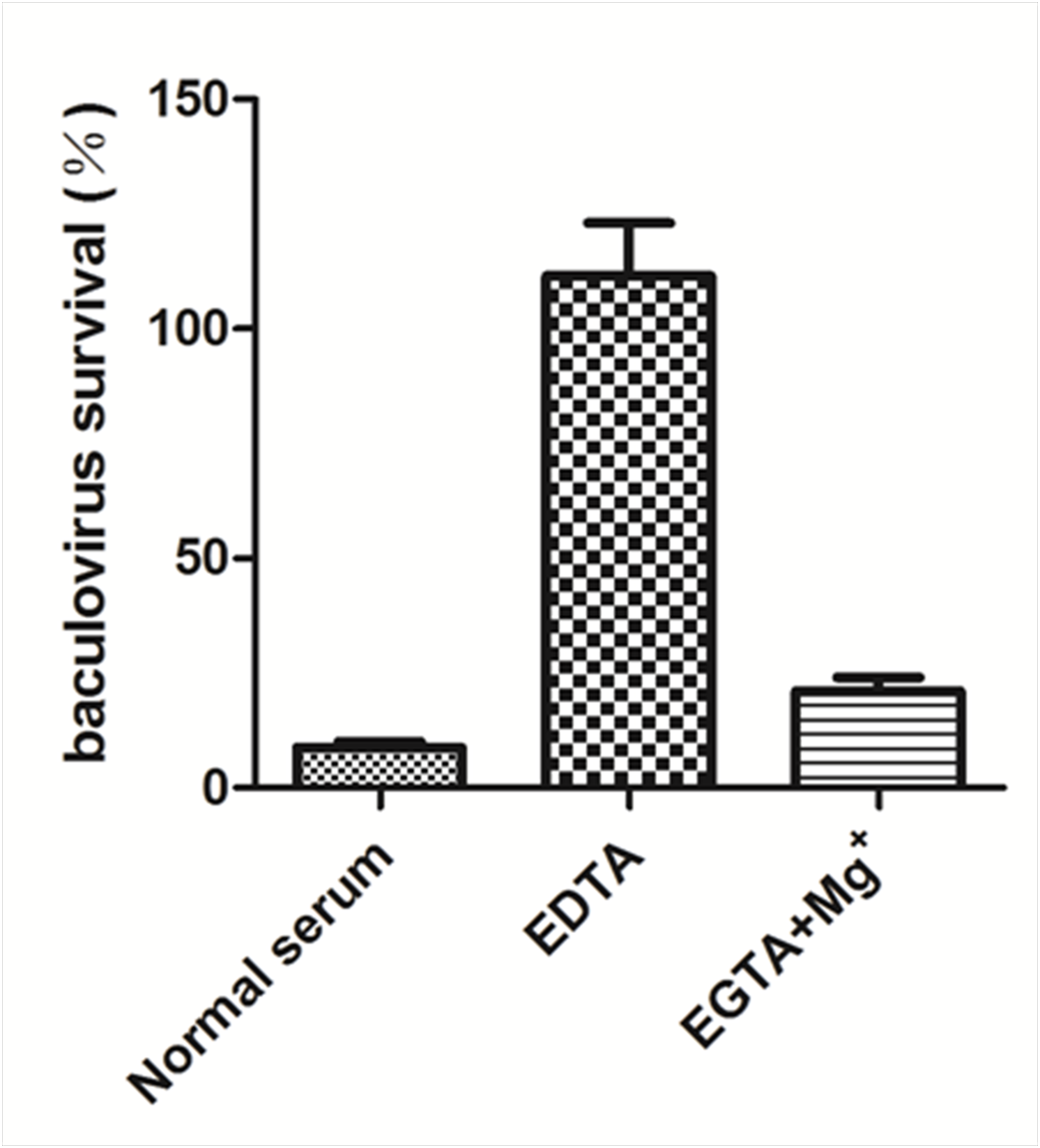
**
