# Supplementary material for: Surface displaying of swine IgG1 Fc enhances baculovirus-vectored vaccine efficacy by facilitating viral complement escape and mammalian cell transduction
Source: Vet Res. 2017 May 12;48:29. doi: 10.1186/s13567-017-0434-5 (PMC5429525; doi:10.1186/s13567-017-0434-5)
Supplement: Supplementary file 2 — Additional file 2. Detection of interaction between IgM in pig serum and recombinant baculoviruses by ELISA. 7 × 10^6^ pfu of baculovirus was coated, and then exposed to pig serum, followed by addition of HRP-conjugated goat-anti-swine IgM. Bars denote the OD450. The assay was performed in triplicate and the data are presented as mean ± SD (**P < 0.05). [file 13567_2017_434_MOESM2_ESM.doc]

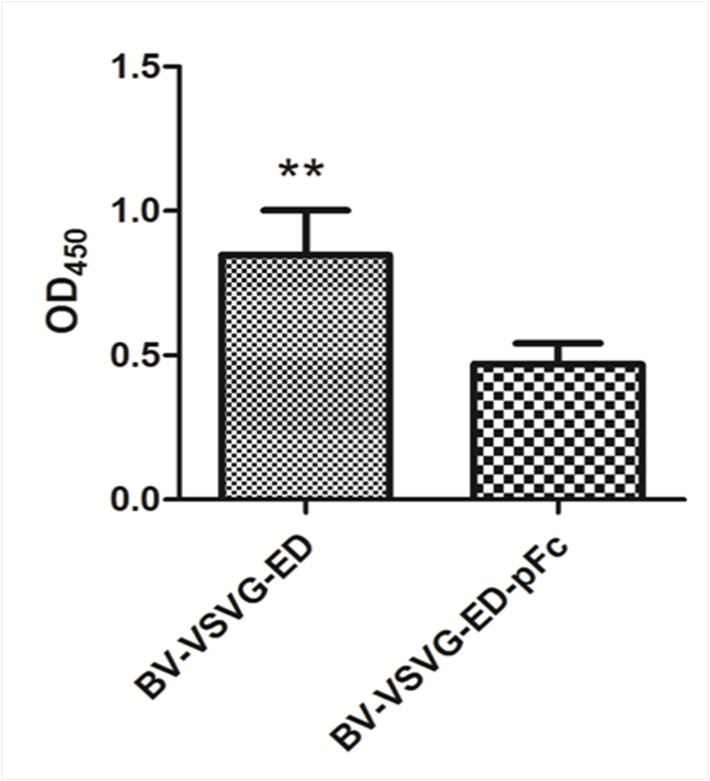
**Fig.S2. Detection of interaction between IgM in pig serum and recombinant baculoviruses by ELISA.**7×10^6^ pfu of baculovirus was coated, and then exposed to pig serum, followed by addition of HRP-conjugated goat-anti-swine IgM. Bars denote the OD_450_. The assay was performed in triplicate and the data are presented as mean ± SD (**, *P* <0.05).
